# A phase II study of dacetuzumab (SGN-40) in patients with relapsed diffuse large B-cell lymphoma (DLBCL) and correlative analyses of patient-specific factors

**DOI:** 10.1186/1756-8722-7-44

**Published:** 2014-06-12

**Authors:** Sven de Vos, Andres Forero-Torres, Stephen M Ansell, Brad Kahl, Bruce D Cheson, Nancy L Bartlett, Richard R Furman, Jane N Winter, Henry Kaplan, John Timmerman, Nancy C Whiting, Jonathan G Drachman, Ranjana Advani

**Affiliations:** 1 David Geffen School of Medicine at UCLA, University of California Los Angeles, 650 Charles E. Young Drive 11-934 Factor Bldg, Los Angeles, CA 90095-1678, USA; 2 University of Alabama at Birmingham, Birmingham, AL, USA; 3 Mayo Clinic Rochester, Rochester, MN, USA; 4 University of Wisconsin, Madison, WI, USA; 5 Georgetown University Hospital, Washington, DC, USA; 6 Washington University School of Medicine, Saint Louis, MO, USA; 7 Weill Medical College of Cornell University, New York, NY, USA; 8 Northwestern University, Chicago, IL, USA; 9 Swedish Cancer Institute, Seattle, WA, USA; 10 Seattle Genetics, Inc, Bothell, WA, USA; 11 Stanford University Medical Center, Stanford, CA, USA

**Keywords:** Diffuse large B-cell lymphoma, DLBCL, Dacetuzumab, CD40

## Abstract

**Background:**

Patients with DLBCL who are ineligible for or have relapsed after aggressive salvage chemotherapy have a poor prognosis. CD40 is expressed on multiple B-cell neoplasms including DLBCL and is a potential target for immunotherapy. Dacetuzumab (SGN-40), a non-blocking, partial agonist, humanized IgG1, anti-CD40 monoclonal antibody, has previously demonstrated anti-lymphoma activity in a phase I study.

**Methods:**

A phase II study was undertaken to evaluate the rate and duration of objective responses and safety of single-agent dacetuzumab in relapsed DLBCL. Forty-six adult patients with relapsed/refractory DLBCL received up to 12 cycles of intravenous dacetuzumab using intrapatient dose-escalation to a target dose of 8 mg/kg/week in an initial 5-week cycle, followed by 4-week cycles of 8 mg/kg/week. Study endpoints included rate and duration of objective responses, safety, survival, pharmacokinetics, immunogenicity, and exploratory correlative studies.

**Results:**

Overall response rate was 9% and disease control rate (complete remission + partial remission + stable disease) was 37%. Common non-hematologic adverse events (AEs) included fatigue, headache, chills, fever, and nausea. The most frequent Grade 3–4 non-hematologic AE was deep venous thrombosis (3 patients). Grade 3–4 lymphopenia (41%), neutropenia (13%), or thrombocytopenia (19%) occurred without associated infection or bleeding. Reversible ocular events, including conjunctivitis and ocular hyperemia, occurred in 8 patients (17%). Patient-specific factors, including Fc-gamma-RIIIa polymorphism, did not appear to correlate with antitumor activity.

**Conclusions:**

Single-agent dacetuzumab has modest activity and manageable toxicity in unselected patients with relapsed DLBCL. Combination regimens and robust methods of patient selection may be necessary for further development.

**Trial registration:**

ClinicalTrials.gov identifier NCT00435916.

## Background

Although the outcome of patients with diffuse large B-cell lymphoma (DLBCL) has improved considerably in the rituximab era [1-3], patients who experience relapse after initial rituximab-containing chemotherapy or after a salvage regimen have a poor prognosis. A significant number of patients are ineligible for aggressive salvage therapy or autologous stem cell transplantation (ASCT) due to comorbid conditions or advanced age. The CORAL study [4] established that patients with DLBCL who relapsed within a year after first-line therapy with R-CHOP have a poor response and survival after ASCT when compared to historical data in patients who relapse after CHOP alone. Thus, better therapeutic options are urgently needed for this population.

CD40 is a member of the TNF-receptor superfamily that functions as a co-stimulatory molecule upon interaction with its ligand CD154 (CD40L) [5]. CD40 is expressed on several types of B-cell neoplasms, including non-Hodgkin lymphoma (NHL), multiple myeloma, and chronic lymphocytic leukemia, making it an attractive potential tumor target for antibody-based cancer therapy [6]. Dacetuzumab (also known as SGN-40) is a humanized IgG1 form of S2C6, a murine anti-human CD40 monoclonal antibody (mAb). In contrast to blocking anti-CD40 mAbs, dacetuzumab does not prevent CD40/CD40L interactions, and the antibody behaves as a partial agonist in vitro. Dacetuzumab has little effect on normal cells in vitro, but in the presence of cross-linking reagents and certain cytokines or growth factors (e.g., interleukin-4 [IL-4]), it enhances the proliferation of responsive cells, such as normal B-cells [7]. In vitro and in vivo studies with the CD40-positive lymphoma lines Ramos and IM-9 demonstrated that dacetuzumab induces apoptosis and inhibits growth of CD40-positive lymphoma lines through direct signal transduction and kills tumor cells through antibody-dependent cellular cytotoxicity (ADCC) and phagocytosis (ADCP). The relevance of both direct and cell-mediated mechanisms was apparent in in vivo models [7]. It has been demonstrated that agonistic CD40 antibodies may mediate antitumor activity through activation of CD40-positive antigen presenting cells, including dendritic cells [8,9].

A phase I study of dacetuzumab was conducted in 50 patients with refractory or recurrent B-cell NHL, including DLBCL, mantle cell lymphoma, follicular lymphoma, small lymphocytic lymphoma, and marginal zone lymphoma [10]. Toxicity appeared acceptable and antitumor activity was observed with 6 objective responses (12%), and 13 patients with stable disease (26%). The overall response rate (ORR) for patients with refractory or recurrent DLBCL was 18% (4 of 21 patients) and provided the rationale for further evaluation of dacetuzumab in this patient population.

The primary objectives of the current study were to determine the ORR, response duration, and safety profile of dacetuzumab in patients with relapsed DLBCL. Secondary objectives included evaluation of survival, pharmacokinetics, immunogenicity, and exploratory analyses to determine whether the antitumor activity of dacetuzumab was associated with FcγR polymorphisms or DLBCL molecular subtypes.

## Methods

### Patients

Adults with a histologically confirmed diagnosis of DLBCL, measurable disease, and an ECOG performance status of ≤2 were eligible. Inclusion criteria included at least 1 prior systemic therapy consisting of combination chemotherapy and rituximab; progression since the most recent therapy; and received standard salvage therapy including ASCT unless deemed ineligible due to age or comorbidities. At the time of enrollment, patients must have been at least 12 weeks from ASCT, 4 weeks from chemotherapy or monoclonal antibody treatment, and 2 weeks from radiation or treatment with immunomodulatory agents. Exclusion criteria included previous diagnosis or treatment for indolent lymphoma; primary refractory disease; progression while receiving salvage therapy; leptomeningeal or central nervous system lymphoma; prior allogeneic transplant; or prior treatment with any anti-CD40 antibody. This was a multicenter study conducted at 10 sites in the United States. Each institution’s Ethics Committee approved the protocol and patients provided written informed consent.

### Study design

For Cycle 1, all patients were treated using an intra-patient dose-escalation schedule to reduce the risk of first-dose toxicities (Table 1) [10]. Subsequent cycles consisted of 4 doses of 8 mg/kg on Days 1, 8, 15, and 22. Patients were medicated before and after dacetuzumab infusion with acetaminophen and diphenhydramine. Steroids were allowed for the prevention and treatment of cytokine release syndrome. Patients were treated with 2 cycles after a complete remission (CR) or until disease progression for a maximum of 12 cycles.

**Table 1:** Schedule and doses of dacetuzumab in Cycle 1 and additional cycles

	**Dacetuzumab dose, mg/kg**						
	**Week 1**	**Week 1**	**Week 2**	**Week 3**	**Week 4**	**Week 5**	**Week 6**
	**Day 1**	**Day 4**	**Day 8**	**Day 15**	**Day 22**	**Day 29**	**Day 36**
Cycle 1	1	2	4	8	8	8	Restage
Cycles 2-12	8	—	8	8	8	Restage	—

Clinical response was assessed by PET-CT scanning after Cycles 1 and 2 and then after even cycles through Cycle 6; CT scans (without PET) were done after Cycles 8, 10, and 12. Responses were scored as CR, partial remission (PR), stable disease (SD), or progressive disease (PD), as defined by the 1999 criteria from the International Workshop on Malignant Lymphoma [11]. Severity of adverse events (AEs) and clinical laboratory values were graded using the National Cancer Institute Common Terminology Criteria for Adverse Events, version 3.0. Serum concentrations of SGN-40 were measured using a validated bridging enzyme-linked immunosorbent assay (ELISA) with a lower limit of quantitation of 0.040 μg/mL. The presence of anti-dacetuzumab (anti-human) antibodies was assessed by Tandem Laboratories (West Trenton, NJ) using a validated enzyme linked immunosorbent assay. Concentrations of circulating cytokines (TNF-alpha, IL-1 beta, IL-6, IL-10, IFN-gamma) were measured by immunofluorescence bead technique at Millipore Corporation (St. Charles, MO). Circulating lymphocyte subsets (CD19+, CD3+, CD3 + CD4+, CD3 + CD8+), monocytes (CD14+), and NK cells (CD16+ CD56+) were measured by flow cytometry at Roswell Park Cancer Institute (Buffalo, NY).

### Correlative studies

A retrospective central pathologic review was conducted by a core lab (Phenopath Laboratories, Seattle, WA). Cases with discordant pathology were adjudicated by R. Gascoyne, MD, University of British Columbia, Vancouver, Canada. Immunohistochemistry was performed for pathologic disease confirmation, DLBCL subtype determination, and CD40 expression. The Hans classification method [12] was used to classify DLBCL subtypes. Immunohistochemistry stains included antibodies against CD20, CD10, Bcl-6, MUM1, Ki67, and CD40. Fcγ receptor IIa and IIIa polymorphism analyses were conducted by W. Weng, MD, Stanford University, Stanford, CA, using the TaqMan technology on an ABI Prism 7900HT Sequence Detector System (Applied Biosystems) as previously described [13].

### Statistics

Analyses of data from this study were primarily descriptive. Missing data were not imputed. Safety and efficacy evaluations were performed on all patients who received any study drug (i.e., safety/modified intent-to-treat [mITT] set). It was expected that approximately 40 patients would be enrolled in the study to ensure at least 35 study-compliant patients who received at least 1 infusion of 8 mg/kg. With 35 patients, assuming an alpha error of 0.05 and a targeted observed response rate of 25%, a one-sided 95% confidence interval would exclude a response rate ≤12%. The Kaplan-Meier method was used to estimate time-to-event endpoints. Maximum observed concentration (Cmax) and minimum observed concentration (Ctrough) were derived from the observed serum concentration data and analyzed descriptively. The effects of FcγR polymorphisms and DLBCL molecular subtype on objective response rate were examined using Fisher’s exact test. Data management and statistical analysis were performed by Seattle Genetics, Inc. All co-authors had access to the primary study data.

## Results

### Patients

A total of 46 patients were enrolled between December 2006 and January 2009. Patients’ characteristics at study entry are summarized in Table 2. The median number of prior therapies was 3 (range: 1–9) and 35% of subjects had previously undergone ASCT.

**Table 2:** Baseline patient characteristics

**Characteristic**	**Median [range] or No. (%)**
Age, years	72 [17–85]
Male gender	28 (61)
ECOG status	
0	16 (35)
1	23 (50)
2	6 (13)
Unknown	1 (2)
Time since diagnosis, years	2.9 [0–21]
Number of prior systemic therapies	3 [1-9]
Prior ASCT	16 (35)
Diagnosis after confirmatory review	
DLBCL	40 (87)
Follicular NHL (grade 2)	3 (7)
Marginal zone	2 (4)
Diffuse large cell NHL, lineage undetermined	1 (2)
DLBCL subtype	
GCB	14 (30)
Non-GCB	14 (30)
Missing	18 (39)
CD40 staining intensity	
0	1 (2)
1+	2 (4)
2+	13 (28)
3+	25 (54)
Not available	5 (11)

ECOG, Eastern Cooperative Oncology Group; ASCT, autologous stem cell transplantation; DLBCL, diffuse large B-cell lymphoma; GCB, germinal center B-cell; NHL, non-Hodgkin lymphoma.

Enrollment was based on a diagnosis of de novo DLBCL at the treating institution, but a pre-planned central pathologic evaluation suggested a discordant diagnosis in 11 patients (24%). Post-hoc adjudication by a third-party pathologist confirmed DLBCL in 40 patients, with other diagnoses in 6 patients (13%) [follicular Grade 2 (3 patients); marginal zone (2 patients); large cell lymphoma of undetermined lineage (1 patient)].

A total of 85 complete or partial cycles of dacetuzumab were administered. Thirty-one patients received one cycle, 7 received 2, 5 received 4, 2 received 6, and one received 8 cycles. The most frequent reasons for discontinuing therapy were progressive disease (n = 32, 70%), followed by adverse events (n = 8, 17%), investigator decision (n = 2, 4%), withdrawn consent (n = 1, 2%), and “other” (n = 3, 7%). Eight patients had a total of 14 dacetuzumab doses delayed for one week due to adverse events; 6 of these delays were in a single patient who had a CR and received a total of 8 treatment cycles. Persistent thrombocytopenia, which had been present (approximately 100,000/mm^3^) at study entry and likely indicative of heavy prior therapy including prior autologous bone marrow transplant, was implicated in 5 of 6 of those dose delays. This patient also skipped a dose due to an adverse event (diarrheal illness). Five patients each had one dose interrupted but subsequently completed due to an infusion reaction (4 patients) or other adverse event (1 patient). Per protocol, there were no reductions in any dacetuzumab dose.

### Safety

Of the 46 patients, 45 (98%) experienced at least one AE during the study and 21 patients (46%) had at least one AE of Grade 3 or higher. Forty-one patients (89%) had at least one AE considered by the treating investigator to be related to dacetuzumab. The most common non-hematologic AEs observed (Table 3A), irrespective of their relationship to dacetuzumab, were Grade 1 or 2 and consisted of fatigue (19 patients [41%]), headache (16 patients [35%]), chills (15 patients [33%]), pyrexia (12 patients [26%]), nausea (11 patients [24%]), diarrhea (9 patients [20%]), and dyspnea (9 patients [20%]). Grade 3–4 hematologic abnormalities were observed in 13 patients (41%; Table 3B).

**Table 3:** **(A) Treatment-emergent non-hematologic adverse events occurring in greater than 10**% **of patients and (B) Grade 3–4 hematologic abnormalities (N** = **46)**

**(A)**					
	**Severity grade, n (%)**				
**Preferred term**	**1**	**2**	**3**	**4**	**Overall (N** = **46)**
Fatigue	9 (20)	8 (17)	2 (4)	0	19 (41)
Headache	14 (30)	2 (4)	0	0	16 (35)
Chills	11 (24)	3 (7)	1 (2)	0	15 (33)
Pyrexia	8 (17)	4 (9)	0	0	12 (26)
Nausea	8 (17)	2 (4)	1 (2)	0	11 (24)
Diarrhoea	8 (17)	1 (2)	0	0	9 (20)
Dyspnoea	4 (9)	5 (11)	0	0	9 (20)
Cough	7 (15)	0	0	0	7 (15)
Back pain	3 (7)	2 (4)	1 (2)	0	6 (13)
Depression	4 (9)	2 (4)	0	0	6 (13)
Musculoskeletal pain	4 (9)	2 (4)	0	0	6 (13)
Abdominal pain	1 (2)	2 (4)	2 (4)	0	5 (11)
Asthenia	2 (4)	2 (4)	1 (2)	0	5 (11)
Constipation	5 (11)	0	0	0	5 (11)
Oedema peripheral	4 (9)	1 (2)	0	0	5 (11)
Oropharyngeal pain	5 (11)	0	0	0	5 (11)
Vomiting	2 (4)	2 (4)	1 (2)	0	5 (11)
**(B)**					
	**Grade 3**		**Grade 4**		
	**n (%)**		**n (%)**		
Lymphopenia	14 (30)		5 (11)		
Neutropenia	5 (11)		1 (2)		
Thrombocytopenia	7 (15)		2 (4)		
Leukopenia	6 (13)		1 (2)		

Eighteen patients (39%) had at least one serious adverse event (SAE) and 8 patients experienced 16 SAEs evaluated as possibly related to dacetuzumab by the treating investigators. These possibly related SAEs included acute pancreatitis and hyperglycemia (2 episodes of each), pneumonia, pancytopenia, fatigue, confusional state, acute renal failure, nausea, dehydration, vomiting, abdominal pain, small intestinal obstruction, febrile neutropenia, and orbital cellulitis (one episode each).

Previous studies have also reported inflammatory eye disorders, elevation of hepatic transaminases, and symptoms of cytokine release syndrome (CRS) with dacetuzumab [10,14]. These events were observed in our study as well, generally within 2 weeks of starting study drug and were for the most part Grade 1 or 2 (Table 4). Eye disorders, including conjunctivitis and ocular hyperemia, experienced by 8 patients (17%) were considered possibly related to dacetuzumab in 6 of these patients. All reports of eye disorders resolved, most within 3 weeks. All were Grade 1 or 2 in severity with the exception of one report of unrelated Grade 3 conjunctival hemorrhage. Additionally, one of these 8 patients had severe orbital cellulitis related to an infection that resulted in enucleation. This patient had a preceding history of ocular H. zoster and developed a streptococcal orbital cellulitis/endophthalmitis, in the setting of a normal neutrophil count and pre-existing B lymphopenia. The treating investigator reported the overall event as possibly related to dacetuzumab. Asymptomatic elevations of hepatic transaminases (AST or ALT) were reported in 18 patients as Grade 1–2 and in one patient as Grade 3. Symptoms potentially reflective of cytokine release syndrome (CRS) were observed in 35 patients (76%) and occurred after the first dose of dacetuzumab in 21 patients (46%). Of these, only one patient had Grade 3 CRS symptoms. A four-fold elevation in serum levels of one or more cytokines was observed on treatment Day 1 in 13 patients and in conjunction with symptoms possibly associated with CRS for 8 of these patients. The only death during treatment was unrelated to dacetuzumab (self-inflicted gunshot wound).

**Table 4:** Ocular toxicities

**Patient ID**	**AE (Preferred term)**	**Study day of onset**	**Duration (Days)**	**Severity**	**Relation to SGN-40**
029-0010	Eye pain	1	4	Grade 1	Possibly
	Eye pain	5	3	Grade 1	Possibly
	Eye pain	8	4	Grade 1	Possibly
029-0011	Eye irritation	1	1	Grade 1	Possibly
	Vision blurred	8	1	Grade 1	Possibly
044-0001	Conjunctivitis	5	14	Grade 2	Probably
	Eye pain	11	11	Grade 2	Possibly
	Eye infection	16	4	Grade 2	Unlikely
	Orbital cellulitis (SAE)	18	4	Grade 3	Possibly
059-0001	Dry eye	16	8	Grade 2	Possibly
066-0010	Conjunctival irritation	16	22	Grade 1	Possibly
	Eye pain	37	36	Grade 1	Possibly
066-0011	Conjunctival hyperaemia	4	11	Grade 2	Possibly
066-0005	Conjunctival hyperaemia	1	56	Grade 1	Unrelated
005-0002	Conjunctival hemorrhage	17	1	Grade 3	Unrelated

Consistent with prior rituximab treatment, all patients entered the study with low numbers of CD19+ cells in peripheral blood (<450 cells/μL), and more than half (57%) had initial CD19+ counts <10 cells/μL. An evaluation of the 9 patients with >100 CD19+ cells/μL at baseline and with post-treatment data available revealed rapid and persistent decreases in B-cells after infusion of dacetuzumab. No other consistent changes in mononuclear cell subsets were noted (data not shown).

Among 32 patients with both pre- and post-treatment data available, no anti-dacetuzumab antibodies were detected. Low titer immune responses could have been missed due to assay interference from dacetuzumab in the sample. Accumulation of dacetuzumab in serum was observed with repeated administration throughout Cycle 1 and subsequent cycles. Peak and trough concentrations increased to means of 204 ± 43.2 and 101 ± 37 μg/mL, respectively, by the end of Cycle 1. Peak concentrations continued to increase at the end of each cycle to 329 ± 84.7 μg/mL for the 3 patients completing the end of Cycle 6, and 465 μg/mL for the one patient completing the end of Cycle 8. The concentration vs. nominal collection time profile was consistent with a biphasic pharmacokinetic pattern (data not shown). Limited sampling precluded calculation of parameters such as clearance and elimination half-life.

### Efficacy

**Figure 1:**
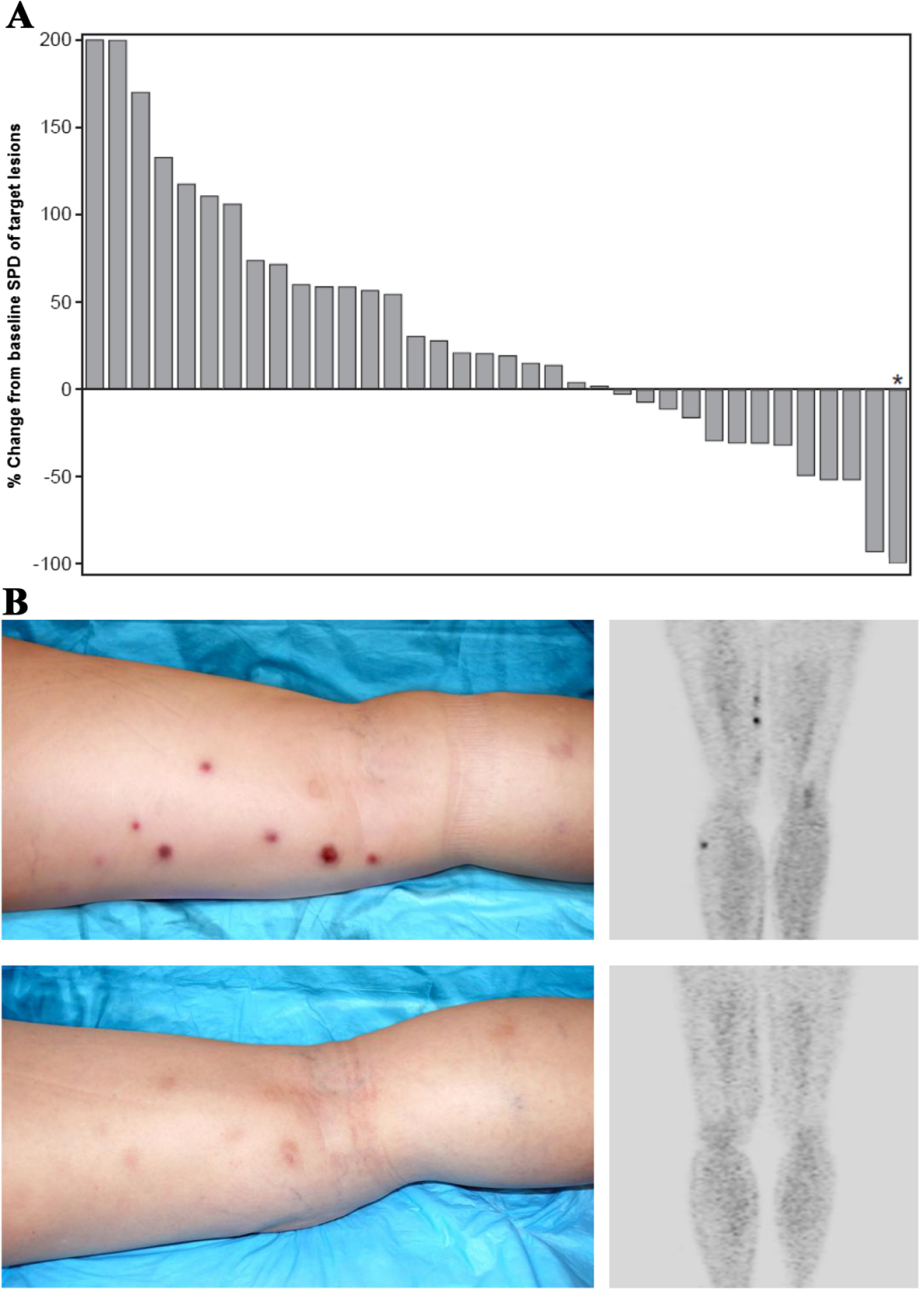
**(A) Maximum reduction in target lesions and (B) complete remission in 80-year-old female with multiple subcutaneous and skin lesions of multifocal primary cutaneous DLBCL leg type.** Waterfall plot of maximum reduction in target lesions for all patients with data before and after treatment with dacetuzumab. Each bar represents change in the sum of the product of perpendicular diameters (SPD) for an individual patient (*denotes pictured case study).

**Figure 2:**
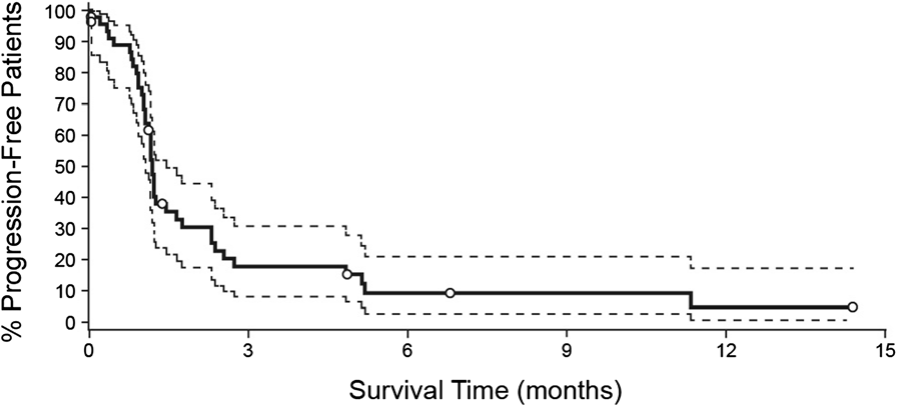
**Kaplan-Meier plot of progression-free survival (PFS) for modified intent-to-treat (mITT) population (solid line).** Dashed lines represent 95% confidence intervals and circles indicate censored patients.

On an intent-to-treat basis, objective responses (CR and PR) were observed in 4 of 46 patients (9%; 2 CR, 2 PR) and 13 patients had SD (28%; Table 5), resulting in a disease control rate of 37% (Figure 1A). The response scores for these patients were consistent between the 1999 and 2007 (revised) response criteria [11,15]. On independent pathology review, 3 of the responders were confirmed to have DLBCL while one partial responder had a diffuse variant of Grade 2 follicular lymphoma. One of the complete responders, with primary cutaneous DLBCL leg type, had multiple right lower extremity skin lesions on physical exam and on PET imaging. This patient had previously failed R-CHOP/radiation therapy, and experienced relapse 4 months after salvage therapy with R-ICE/radioimmunotherapy. After treatment with dacetuzumab, the patient’s lesions completely resolved both clinically and radiologically (Figure 1B). The objective response duration was over one year, after which recurrent DLBCL was documented by biopsy. A second course of dacetuzumab (6 cycles) was then administered under a compassionate use protocol, resulting in a near CR; this second response was ongoing after more than 20 months. The other complete responder, who had received 4 prior chemotherapy regimens including ASCT plus radiotherapy, had complete radiographic resolution of multiple nodes of which the largest was 7.7 × 3.6 cm at baseline. This patient had an objective response duration of 262 days. The 2 PRs lasted 78 and 78+ days; the latter case was that of a patient with Grade 2 follicular lymphoma who had enrolled on study due to inability to tolerate intensive salvage therapy, and after achieving a PR with 4 cycles of dacetuzumab, proceeded to ASCT. Duration of SD exceeded 120 days in 3 patients. Overall, the median progression-free survival was 36 days (range, 1-438+ days, Figure 2).

**Table 5:** **Investigator assessment of best clinical response**^**a**^

**Best clinical response**	**All patients**
	**(N** = **46), n (%)**
CR^b^	2 (4)
PR	2 (4)
SD	13 (28)
PD	26 (57)
Unknown^c^	3 (7)

^a^Per response criteria in Cheson et al. [11].

^b^Both CRs were confirmed.

^c^No post-baseline evaluations available.

### Exploratory correlative evaluations

There was no significant correlation between responses or disease control and FcγR IIa and FcγR IIIa polymorphisms, DLBCL molecular subtype, CD40 expression level, or prior ASCT (Table 6).

**Table 6:** Subgroup analyses

	**Available data**	**Number of responders**	**p-value**^**a**^	**Number with disease control**	**p-value**^**a**^
	**n (%)**	**(CR + PR)**		**(CR + PR + SD)**	
**FcγRIIa polymorphism** (N = 27)			0.12		0.19
H/R	13 (48)	0		4	
H/H	7 (26)	1		2	
R/R	7 (26)	2		5	
**FcγRIIIa polymorphism** (N = 32)			>0.99		0.72
V/F	13 (41)	2		6	
F/F	12 (38)	1		4	
V/V	7 (22)	1		2	
**DLBCL molecular subtype** (N = 27)			0.66		>0.99
GCB	14 (52)	1		4	
ABC	11 (41)	2		4	
Other	2 (7)	0		1	
**CD40 staining intensity** (N = 38)			0.17		0.24
3+	22 (58)	3		8	
2+	13 (34)	0		7	
1+	2 (5)	1		2	
0	1 (3)	0		0	
**CD40 expression** (N = 38)			0.45		>0.99
≥2+ and ≥80% positive	33 (87)	3		15	
<2+ OR <80% positive	5 (13)	1		2	
**Prior ASCT** (N = 43)			0.62		0.75
Yes	16 (37)	2		7	
No	27 (63)	2		10	

^a^Fisher’s exact test.

## Discussion

This study confirms and extends prior clinical observations of the anti-CD40 antibody dacetuzumab administered to patients with non-Hodgkin lymphoma [10]. Overall, the safety profile for dacetuzumab was acceptable and manageable in this patient population. Consistent with prior experience and the known cellular target of dacetuzumab, there was evidence of dacetuzumab-related B-cell depletion. Baseline lymphopenia likely reflects, in large part, extensive prior treatment of the study patients with multiple myelosuppressive regimens and rituximab. Total lymphocyte depletion > Grade 3 was identified in 18% of patients, but did not result in high rates of clinical infections. A notable exception was a single case of bacterial orbital cellulitis requiring enucleation after ocular inflammation. Noninfectious ocular inflammation has been potentially attributed to reports of CD40 expression on conjunctival tissue, and mild conjunctival symptoms reported 12-21% of patients in phase I trials with dacetuzumab [10,14,16]. Consistent with prior phase I experience, mild symptoms related to CRS were noted, easily managed with premedications and did not compromise the ability to administer dacetuzumab. The rate of elevated asymptomatic hepatic transaminase values was also consistent with prior clinical experience. The observed dacetuzumab serum concentrations and the absence of host antibody responses also mirrored prior clinical experience.

In this study, restricted to a heavily pretreated DLBCL population, a 9% objective response rate was observed with a disease control rate of 37% (CR + PR + SD). In a retrospective pathology review, 1 patient with a PR was found to have Grade 2 follicular lymphoma. Therefore, the ORR for confirmed DLBCL patients in this study was 7%. This trial confirms the limited single-agent antitumor activity of dacetuzumab in aggressive NHL reported in the phase I trial [10]. Though infrequent, the individual responses seen in the present trial were robust. Most notable was a durable CR that was attained in a case of multifocal primary cutaneous DLBCL leg type, a distinct DLBCL subtype with characteristic molecular features and an unfavorable prognosis [17,18], that had relapsed shortly after failure of aggressive salvage therapy. Moreover, at a subsequent relapse, the patient’s disease again responded nearly completely to re-treatment with dacetuzumab. A second CR was obtained in a patient who had previously failed therapy with multiple regimens, including ASCT. The individual responses observed suggest that selected patients with DLBCL could benefit from treatment with dacetuzumab.

DLBCL is a notably heterogeneous group of lymphomas with different pathogeneses, driver mutations, and pathway dependencies. CD40-targeting is a precise therapeutic approach. It is not surprising that only a subset of unselected patients with DLBCL benefited from treatment with dacetuzumab. Predictive biomarkers may allow enriching the patient population likely to respond. However, predetermined correlative studies in the present trial did not identify factors that could predict clinical benefit. Specifically, the uniformity and intensity of CD40 expression, DLBCL subtype, and FcγR polymorphism did not correlate with response or stable disease.

In contrast, Burington et al. [19] examined the activation status of the CD40 pathway, and identified and validated an associated 15-gene qRT-PCR signature generated from preclinical models of NHL. The 15-gene signature predicted outcomes following CD40 pathway stimulation and suggested that CD40 activation status was a significant factor predicting response. It was applied to patient samples from two separate dacetuzumab monotherapy studies. Data from 28 of the 39 patient samples used in the training set for the clinical exploratory work were collected as part of the present phase II trial. A low baseline CD40 pathway activity correlated with high sensitivity to dacetuzumab and a high baseline CD40 pathway activity correlated with resistance to dacetuzumab [19]. The mechanism of resistance to dacetuzumab might be the selective activation of the noncanonical NF-κB signaling pathway in constitutively active CD40 receptors that could promote cell survival by shifting the balance to an anti-apoptotic response [20]. A limitation of this signature is that it only relates to the CD40 signaling mechanism of action of SGN-40 and does not take into account other potential mechanisms, such as effector functions (e.g., ADCC) or activation of immune cells (APCs). Therefore, additional work is needed to identify which patient-specific characteristics may be associated with response to dacetuzumab.

Preclinical studies with dacetuzumab have shown synergy when combined with rituximab or combination chemotherapy in animal models [21,22]. This has led to additional trials of dacetuzumab in combination with standard agents. Activity in relapsed DLBCL was reported in a single-arm Phase Ib trial of dacetuzumab in combination with rituximab and gemcitabine, showing an ORR of 47% (14 of 30 patients) in relapsed or refractory DLBCL [23]. Separately, a randomized phase IIb trial of rituximab, ifosfamide, carboplatin, and etoposide (R-ICE) with or without dacetuzumab was conducted in patients who previously failed R-CHOP. Although the trial was stopped early due to failure to increase the CR rate, extended follow-up demonstrated a trend toward increased survival for those patients who were randomized to receive dacetuzumab [24]. These studies suggest that dacetuzumab may be beneficial when combined with active regimens. However, the selection criteria to identify these patients have not been identified. Any future development of dacetuzumab should evaluate the multiple potential mechanisms of action that have been documented preclinically, including ADCC, ADCP, induction of apoptosis by signaling through CD40 on malignant cells, and immunomodulation by activation of CD40-expressing immune cells.

## Conclusions

In summary, this phase II trial of single-agent dacetuzumab confirms that the partial-agonist anti-CD40 is well tolerated and has modest activity in heavily pretreated patients with DLBCL. Because DLBCL is a heterogeneous disease, the challenges ahead are to identify the subset of patients in whom response could be predicted and to develop rationally designed clinical trials.

## Competing interests

All authors received research funding from Seattle Genetics, Inc. with the exception of NCW and JGD who are employees of and have equity ownership in Seattle Genetics, Inc. In addition, NB is a consultant for Seattle Genetics, Inc.

## Authors’ contributions

SD, AFT, SMA, BK, BDC, NLB, RRF, JNW, HK, JT, and RA were investigators in the trial, acquired data for the analysis, and participated in the analysis of the data. NCW and JGD participated in the design of the trial, analysis of the data, and drafting of the manuscript. All authors participated in revising the manuscript and gave final approval for the paper.
